# That Sepia Case*See *Progressive Homœopathy*, by Dr. Lippe, December number (1886), page 429.—Eds.

**Published:** 1887-02

**Authors:** D. C. McLaren

**Affiliations:** Brantford, Ontario


### THAT SEPIA CASE.*

D. C. McLaren, M. D., Brantford, Ontario.

Over eighteen months ago I had to prescribe for a chronic headache, accompanied with fullness of the neck ; the neck-band was frequently loosened during an attack. *Lachesis* proved of no value whatever, nor either of several other drugs. Finally, upon analyzing the case, *Sepia* came to the surface as covering more symptoms than any other drug, and it effected a prompt cure. Slight returns of the headache have been promptly checked by *Sepia*, but that fullness of the neck has never been felt since the first dose. The symptom, having been so distinctly verified by Dr. Lippe, should now rank as a keynote. Bœnninghausen's *Relations* is a sealed book to most homœopaths, but a few more cases like that recorded by Dr. Lippe would teach us how to use it successfully.

* See *Progressive Homœopathy*, by Dr. Lippe, December number (1886), page 429.—Eds.

